# Prognostic Value of the Conversion to a Shockable Rhythm in Out-of-Hospital Cardiac Arrest Patients with Initial Non-Shockable Rhythm

**DOI:** 10.3390/jcm8050644

**Published:** 2019-05-09

**Authors:** Kap Su Han, Sung Woo Lee, Eui Jung Lee, Su Jin Kim

**Affiliations:** Department of Emergency Medicine, College of Medicine, Korea University, Inchon-ro 73, Seongbuk-gu, Seoul 02841, Korea; hanks96@hanmail.net (K.S.H.); kuedlee@korea.ac.kr (S.W.L.); ironlyj@gmail.com (E.J.L.)

**Keywords:** out-of-hospital cardiac arrest, conversion, return of spontaneous circulation, pre-hospital

## Abstract

In patients with out-of-hospital cardiac arrest (OHCA) with an initial non-shockable rhythm, the prognostic significance of conversion to a shockable rhythm (or hereafter “conversion”) during resuscitation remains unclear. We investigated whether conversion is associated with good neurologic outcome. We included patients with OHCA with medical causes and an initial non-shockable rhythm by using the national OHCA surveillance cohort database of the Korea Centers for Disease Control and Prevention for 2012~2016. The primary outcome was good neurologic outcome at hospital discharge. Of 85,602 patients with an initial non-shockable rhythm, 17.9% experienced conversion. Patients with and those without conversion had good neurologic outcome rates of 3.2% and 1.0%, respectively (*p* < 0.001). In multiple regression analysis, conversion was associated with good neurologic outcome (adjusted odds ratio (OR) 2.604; 95% confidence interval (CI) 2.248–3.015) in the patients with an initial non-shockable rhythm, and had the association with good neurologic outcome (adjusted OR 3.972, 95% CI 3.167–4.983) in unwitnessed patients by emergency medical services (EMS) without pre-hospital return of spontaneous circulation (ROSC) among the population. In patients with OHCA with an initial non-shockable rhythm, even if with unwitnessed arrest by EMS and no pre-hospital ROSC, continuing resuscitation needs to be considered if conversion to a shockable rhythm occurred.

## 1. Introduction

Patients with out-of-hospital cardiac arrest (OHCA) have good neurological prognoses when the initial rhythm is shockable [1,2,3]. Those patients with an initial non-shockable rhythm who during cardiopulmonary resuscitation (CPR) convert to a shockable rhythm may also have a good prognosis [4]. However, the prognostic significance of conversion from a non-shockable to a shockable rhythm during the course of resuscitation remains unclear. Thomas et al. reported that conversion to shockable rhythm during resuscitation of OHCA patients did not affect survival [5]. Conversely, Fukuda et al. reported that the conversion from non-shockable to shockable rhythm was one of the predictors for good prognosis in unwitnessed OHCA patients [6].

In OHCA patients not witnessed by emergency medical services (EMS), initial non-shockable rhythm and no return of spontaneous circulation (ROSC) in the pre-hospital setting, conversion to a shockable rhythm is an important determinant in maintaining resuscitation due to the Universal Termination of Resuscitation (TOR) Guidelines [7]. A prerequisite for TOR are: (1) cardiac arrest not witnessed by EMS. (2) no ROSC. (3) no shock was delivered [7,8,9].

In this study, we investigated whether conversion to a shockable rhythm is associated with good neurologic outcome according to the pre-hospital ROSC status and the state of being witnessed by an EMS provider in the patients with initial non-shockable rhythm, based on the Korean national OHCA Surveillance database [10].

## 2. Experimental Section

### 2.1. Study Design and Data Source

This study was based on a nationwide Korean papulation observational cohort—Out-of-Hospital Cardiac Arrest Surveillance (OHCA Surveillance)—of patients in whom resuscitation was attempted after OHCA [10]. We conducted a secondary analysis of the OHCA Surveillance database. The OHCA Surveillance system was initiated in 2008 and is conducted by the Korea Centers for Disease Control and Prevention (KCDC) [10,11]. The registry is based on Utstein-style guidelines [12]. The registry included data on demographic characteristics; whether the arrest was witnessed by an EMS provider or a lay rescuer; the presumed cause of arrest; the incidence of suspected or confirmed trauma; the presence of bystander CPR; the cardiac rhythm initially documented; the presence of a do-not-attempt-resuscitation (DNAR) order or terminal illness; the achievement of pre-hospital ROSC, defined as the return of spontaneous circulation at ED admission; the ROSC; the presence of therapeutic hypothermia or targeted temperature management; the presence of coronary angiography; and the Glasgow–Pittsburgh cerebral performance category (CPC) score at discharge.

The Korean EMS is a single tiered, government-based system covering approximately 50 million residents. EMS personnel cannot declare death at the scene or terminate CPR unless there is a ROSC. Therefore, all patients with OHCA are transported to the emergency department (ED), after which EMS personnel report the patient’s information and CPR related factors—location of arrest; the presence of witnesses; the rhythm initially documented; the presence of bystander CPR; prehospital defibrillation—through the electrical server of the national emergency management agency. KCDC researchers then visit the medical facilities to which the patients were transferred, review medical records, and conduct surveys according to a structured survey form. All patients with OHCA in Korea are included in this surveillance.

### 2.2. Selection of Study Patients

Data from January 2012 to December 2016 were extracted from the database. Patients aged ≥18 years with OHCA due to medical causes, with an initial non-shockable rhythm, and in whom resuscitation was attempted by EMS personnel, were included. Patients who were transferred from the ED to other facilities and those whose families refused further resuscitation were excluded. Patients with an initial shockable rhythm, including ventricular fibrillation and pulseless ventricular tachycardia, were also excluded. Conversion to a shockable rhythm was judged by the presence of electrical shock delivery during CPR in patients with OHCA with an initial non-shockable rhythm. The term “conversion to shockable rhythm” was simply defined as conversion. The conversion group comprised patients with electrical shock delivery during CPR and the non-conversion group comprised those without electrical shock delivery during CPR. We analyzed the effect of conversion to a shockable rhythm on the outcome.

### 2.3. Outcome and Data Analysis

The primary outcome was a neurologically favorable outcome at hospital discharge, defined as a CPC score of 1 or 2. The CPC score was categorized by the medical record reviewer based on the discharge summary. The secondary outcome was survival at hospital discharge.

The basic characteristics and CPR-related variables were compared between the good neurologic outcome group and the poor neurologic outcome group. Multiple logistic regression analyses for outcomes were performed to assess the associations between conversion to a shockable rhythm and good neurologic outcomes after adjusting for selected covariates including age, sex, presence of a witness, initial non-shockable rhythm type (pulseless electrical activity, PEA or asystole), attempted bystander resuscitation and public place of cardiac arrest. Multiple regression analysis was performed according to pre-hospital ROSC and being witnessed by an EMS provider.

### 2.4. Statistical Analysis

Values are presented as mean ± standard deviation or median (interquartile ranges, IQR) for continuous variables and number (%) for categorical variables. Continuous variables were compared using unpaired Student’s t-test or analysis of variance. When the normality assumption was violated (Kolmogorov–Smirnov normality test), continuous variables were compared using Mann–Whitney U non-parametric tests. Categorical variables were compared using chi-square or Fisher’s exact tests. We analyzed the effect of conversion to a shockable rhythm on outcomes by using multiple logistic regression analysis. All statistical analyses were carried out using IBM SPSS Statistics for Windows, version 20.0 (IBM Corp., Armonk, NY, USA). Two-tailed p-values of <0.05 were considered statistically significant.

## 3. Results

### 3.1. Main Categories and Outcomes of the Study Population

**Figure 1:**
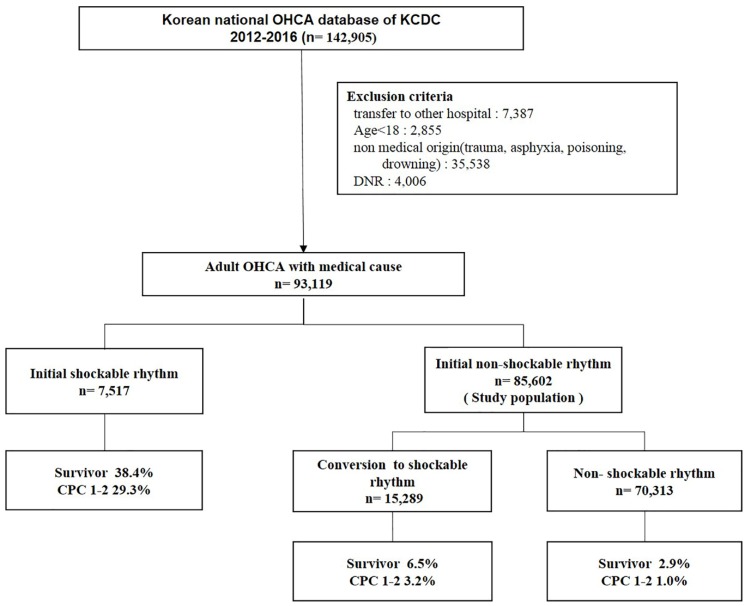
Study population and clinical outcome. OHCA, out-of-hospital cardiac arrest; KCDC, Korea Centers for Disease Control and Prevention; DNR, do not resuscitate; ED, emergency department; CPC, cerebral performance category.

In total, 142,095 patients with OHCA were included in the OHCA Surveillance registry during the study period. This study included 85,602 OHCA patients with an initial non-shockable rhythm, of whom 15,289 (17.9%) experienced conversion to a shockable rhythm during resuscitation (conversion group) and 70,313 (82.1%) maintained a non-shockable rhythm (non-conversion group) (Figure 1).

In patients with OHCA and an initial non-shockable rhythm, the overall survival rate at hospital discharge was 3.5% and the rate of good neurologic outcome at hospital discharge was 1.2%.

The rates of survival to discharge in the conversion and non-conversion groups were 6.5% and 2.9%, respectively (*p* < 0.001) and the rates of good neurologic outcome at hospital discharge were 3.2% and 1.0%, respectively (*p* < 0.001) (Figure 1).

### 3.2. Comparison of Basic Characteristics according to Good Neurologic Outcome and Multiple Logistic Regression Analysis for Outcomes

Table 1 shows the basic characteristics and CPR variables, stratified according to good neurologic outcome (CPC 1–2). Patients with CPC 1–2 were significantly younger; more likely to be male; and have a witnessed arrest, received bystander CPR, and experienced OHCA in a public place and have conversion to shockable rhythm compared to those with CPC 3–5 (Table 1). There was no significant difference in the proportion of presumed cardiac origin between patients with CPC1–2 and CPC 3–5 (Table 1).

After adjustment for known confounders, multiple logistic regression revealed that younger age, male sex, presence of a witness, attempted bystander CPR, presence of ROSC in the pre-hospital stage and presence of conversion were independent predictors of survival and good neurologic outcomes at hospital discharge. The adjusted odds ratios (ORs) of pre-hospital ROSC for good neurologic outcomes were 72.777 (95% confidence interval (CI) 63.182–83.828) (Table 2). The adjusted ORs of conversion for survival and good neurologic outcomes were 2.604 (95% CI 2.248–3.015) (Table 2).

The conversion group were significantly younger; more likely to be male; and have a witnessed arrest, received bystander CPR, and experienced OHCA in a public place compared to the non-conversion group (Table 3).

### 3.3. Outcomes and Logistic Regression Analysis according to Pre-Hospital Return of Spontaneous Circulation (ROSC)

Of the 85,602 study patients, 1949 (2.3%) patients had ROSC at the pre-hospital stage and 83,653 (97.7%) did not. Of the patients with pre-hospital ROSC, 37.7% had good neurologic outcomes; however, only 0.6% of those without pre-hospital ROSC had good neurologic outcomes (*p* < 0.001). Among the patients with an initial non-shockable rhythm, the prognosis differed between those with and without ROSC in pre-hospital stage; thus we analyzes the patients according to pre-hospital ROSC status. Regardless of the pre-hospital ROSC status, patients with CPC1–2 were significantly younger and more likely to be male to have had a witnessed arrest, to have received bystander CPR, to have experienced OHCA in a public place, and to have conversion to shockable rhythm more often than patients with CPC 3–5 (Table 4).

In patients with pre-hospital ROSC, multiple logistic regression revealed that younger age (OR for 1-year increment 0.968, 95% CI 0.962–0.975), male sex (OR 1.302, 95% CI 1.045–1.623), witnessed arrest (OR 1.872, 95% CI 1.479–2.369), arrest location at public place (OR 1.462, 95% CI 1.145–1.867), bystander CPR (OR 1.477, 95% CI 1.118–1.885), and conversion to shockable rhythm (OR 2.325, 95% CI 1.821–2.935) were associated with good neurologic outcome (Table 5). In patients without pre-hospital ROSC, multiple logistic regression revealed that younger age (OR for 1-year increment 0.954, 95% CI 0.948–0.959), witnessed arrest (OR 5.313, 95% CI 4.203–6.716), initial rhythm (PEA) (OR 1.469, 95% CI 1.122–1.922), arrest location at public place (OR 1.364, 95% CI 1.091–1.704), bystander CPR (OR 1.406, 95% CI 1.135–1.741), and conversion to a shockable rhythm (OR 2.607, 95% CI 2.170–3.131) were associated with good neurologic outcome but male sex was not (Table 5).

### 3.4. Outcomes and Logistic Regression Analysis according to the State of being by an Emergency Medical Services (EMS) Provider in Patients without Pre-Hospital ROSC

**Figure 2:**
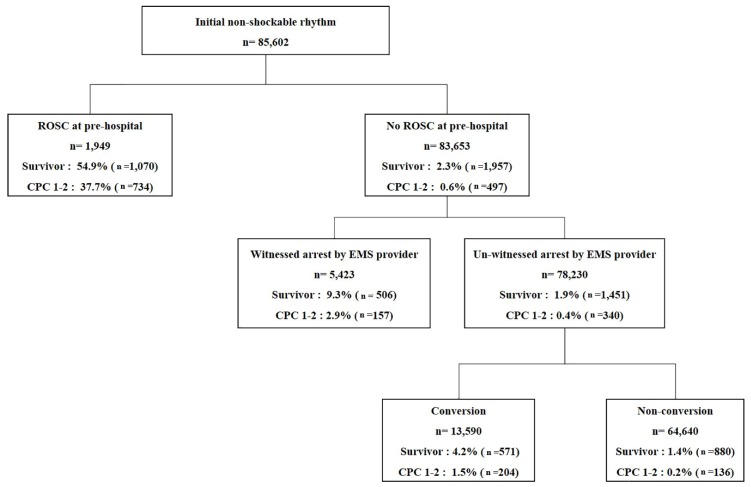
Outcomes according to pre-hospital ROSC and the state of being witnessed by an EMS provider. EMS, emergency medical services; CPC, cerebral performance category.

In patients without pre-hospital ROSC, the rates of survival to discharge for the group witnessed by EMS provider and the group not witnessed by an EMS provider were 9.3% and 2.3%, respectively (*p* < 0.001) and the rates of good neurologic outcome at hospital discharge were 2.9% and 0.4%, respectively (*p* < 0.001) (Figure 2).

In patients without pre-hospital ROSC and un-witnessed by an EMS provider, the rates of survival to discharge in the conversion and non-conversion groups were 4.2% and 1.4%, respectively (*p* < 0.001) and the rates of good neurologic outcome at hospital discharge were 1.5% and 0.2%, respectively (*p* < 0.001) (Figure 2).

In patients without pre-hospital ROSC and un-witnessed by an EMS provider, after adjustment for known confounders, multiple logistic regression revealed that conversion to a shockable rhythm (OR 3.972, 95% CI 3.167–4.983) was associated with good neurologic outcome (Table 6). In patients without pre-hospital ROSC and witnessed by an EMS provider, after adjustment for known confounders, multiple logistic regression revealed that conversion to a shockable rhythm (OR 1.052, 95% CI 0.732–1.511) was not associated with good neurologic outcome (Table 6).

## 4. Discussion

In this study, conversion to shockable rhythm among OHCA patients with an initial non-shockable rhythm was associated with good neurologic outcomes. Regardless of the prehospital ROSC status, conversion to a shockable rhythm was associated with good neurologic outcomes. In OHCA patients without ROSC at pre-hospital stage and un-witnessed by an EMS provider, conversion to a shockable rhythm was associated with good neurologic outcome.

In previous studies, conversion to shockable rhythm during resuscitation was associated with good prognosis; however, the results were not consistent. The first report on this topic was published in 2007 by Hallstrom et al. They reported that the survival-to-discharge rates were lower in patients with conversion to shockable rhythm than in those without this conversion [13]. In contrast, Olasveengen et al., Kajino et al., and Herlitz et al. showed that conversion to a shockable rhythm was associated with better outcomes in patients with an initial non-shockable rhythm [14,15,16]. In 2013, Thomas et al. conducted a secondary analysis of data in the epidemiological registry (Epistry-cardiac arrest) in the United States and Canada [5]. They found that conversion to a shockable rhythm was not associated with survival to hospital discharge. Conversely, in several subsequent studies using cohorts of patients with cardiac arrest in Japan, North America, Denmark, and Asia, conversion to a shockable rhythm was associated with good outcomes [6,17,18,19]. In our study, conversion to a shockable rhythm was also associated with good neurologic outcome. There is no clear mechanism to explain why conversion to a shockable rhythm is associated with a better prognosis. Our hypothesis is that CPR can supply oxygenated blood to the heart and, then, if the conversion occurs from non-shockable to shockable rhythm, it can be assumed that there is still electrical activity in the heart compared to when the conversion is not occurring. However, there was no study for showing clear pathophysiological evidence of conversion to a shockable rhythm, so further studies such as animal experiments are needed to know the mechanism.

As noted above, the majority of studies reported that conversion to a shockable rhythm were associated with good outcomes, but some of these studies reported other results, possibly because of the difference in the target patient group and differences in the EMS protocol depending on the region. The study population differed in terms of the percentage of patients who had conversion to a shockable rhythm and the ratio of patients with the PEA to those with asystole [4]. Zheng et al. reported that conversion to shockable rhythm was not associated with better outcomes in patients with initial PEA, whereas conversion was associated with good outcomes in those with initial asystole [19]. Funada et al. reported age-specific differences in prognostic significance of rhythm conversion. They found that subsequent shock delivery is associated with good neurologic outcomes in patients aged 18–74 years but not in those aged ≥75 years [20]. In addition, there may be differences in results because the transfer protocol and the treatment guidelines in the field are different in each region. In Asia in particular, almost all patients with cardiac arrest are sent to the hospital without suspending resuscitation in the field, which may be different from the situation in the Western countries. Four studies conducted in Asia have shown that conversion to shockable rhythm was associated with good prognosis.

We also analyzed the effect of conversion to a shockable rhythm according to pre-hospital ROSC, which is considered as the most crucial factor for survival and good neurologic outcome in patients with OHCA [6]. Wampler et al. reported that survival with pre-hospital ROSC was 17.2% (154 of 894) and without pre-hospital ROSC was 0.69% (11 of 1589) in patients with non-traumatic OHCA [21]. Pre-hospital ROSC is considered as outcome of resuscitation care as well as the indicator for good outcome. Our study also showed that pre-hospital ROSC with the highest odds ratio of 72.777 (95% CI 63.182–83.828) for good neurologic outcome in study population. Among patients with an initial non-shockable rhythm, 37.7% with pre-hospital ROSC had good neurologic outcomes compared with only 0.6% of patients without pre-hospital ROSC. Despite advances in cardiopulmonary resuscitation guidelines and post-arrest care, OHCA patients who have not ROSC in the pre-hospital stage still have a poor prognosis. However, it is difficult to decide to terminate CPR in patients with cardiac arrest. Currently, the guideline used for the termination of CPR is the universal TOR guideline [7]. The TOR guideline consists of three requirements: un-witnessed by the EMS provider, non-delivery of shock, the absence of ROSC. If the three criteria are met, discontinuing resuscitation may be considered. The reason for the use of this guideline is that it is difficult to expect a good prognosis if the three requirements are met in patient with OHCA. Drennan et al. reported that the survival-to-discharge rate was 0.7% and the good neurologic outcome rate was 0.3% in the TOR termination group, which satisfied the aforementioned criteria [8]. In our study, the rate of good neurological outcomes in patients who met all three TOR criteria was 0.2%.

Being witnessed by EMS personnel and the type of the initial arrest rhythm can be quickly judged from the initial assessment of cardiac arrest. Therefore, in OHCA patients with an initial non-shockable rhythm and not witnessed by an EMS provider, if pre-hospital ROSC is not observed, conversion to a shockable rhythm can be a factor that determines continuation of resuscitation. In our study, conversion to a shockable rhythm was associated with good neurologic outcome in patients without pre-hospital ROSC and un-witnessed by an EMS provider. However, this does not mean that resuscitation should be stopped according to the TOR guidelines. Because the EMS system differs in each region and different treatments are performed in the field, the guidelines for TOR cannot be uniformly applied. Further, there are many patients who are eligible for the universal TOR criteria although their prognosis is poor; thus there are many patients with good neurologic outcome who are eligible for the universal TOR. In our study, because the proportion of patients who are eligible for the universal TOR criteria was high, 136 patients who met the universal TOR criteria had good neurologic outcome. Therefore, uniformly using the TOR guidelines in all patients with cardiac arrest is not recommended. The application of the universal TOR guidelines requires clinical considerations.

In OHCA patients with a non-cardiac medical origin with an initial non-shockable rhythm, conversion to shockable rhythm was not associated with good neurologic outcomes. Varvarousi et al. reported that the conversion group showed lower ROSC rate than the non-conversion group in an animal asphyxial cardiac arrest study [22].

This study has several limitations. First, we classified patients only according to their initial rhythm and did not analyze the rhythm just before the conversion to a shockable rhythm. For accurate analysis, the rhythm before conversion should be analyzed; however, our study examined the effect of conversion to a shockable rhythm according to the initial rhythm. In addition, if the analysis was performed according to the defibrillation status after conversion to a shockable rhythm, the effect of defibrillation will be known. However, according to the current guideline, defibrillation should be performed when shockable rhythm is observed. Second, this study did not consider the EMS response time. The prognosis may vary depending on the EMS response time. However, the research data we collected did not include this information. Future studies should collect this important detail. Third, we did not consider the time when the conversion occurred. In previous studies, conversion was effective when the conversion time was <20 min [23,24]. Although our study did not include the time to conversion to a shockable rhythm, conversion both before and after hospital arrival was effective; however, the OR was higher when conversion occurred before ED admission (OR 2.915, 95% CI 2.427–3.500) rather than after (OR 2.290, 95% CI 1.881–2.789). Therefore, early rhythm conversion was likely more effective; however, further studies are required to confirm this assumption.

## 5. Conclusions

In the nationwide OHCA database cohort, OHCA patients with an initial non-shockable rhythm in whom conversion to shockable rhythm occurred showed better neurologic outcomes than those without conversion. In patients without pre-hospital ROSC and un-witnessed by an EMS provider, subsequent rhythm conversion was associated with good neurologic prognosis. In OHCA patients with an initial non-shockable rhythm, even if there was no ROSC in the pre-hospital stage and the arrest was not witnessed by an EMS provider, continuation of resuscitation can be considered if conversion to a shockable rhythm occurred.

## Figures and Tables

**Table 1:** Characteristics and resuscitation variables according to good neurologic outcome in patients with an initial non-shockable rhythm.

Variable	All (*n* = 85,602)	CPC 1–2 (*n* = 1231, 1.4%)	CPC 3–5 (*n* = 84,371, 98.6%)	*p*-Value
Age (year), median (IQR)	73(60–81)	56 (48–66)	73(60–81)	<0.001
Age group (years)				<0.001
18~44	5282 (6.2)	198 (16.1)	5084 (6.0)	
45~64	21,828 (25.5)	681 (55.3)	21,147 (25.1)	
65~74	18,763 (24.9)	216 (17.5)	18,547 (22.0)	
≥75	39,729 (46.4)	136 (11.0)	39,593 (46.9)	
Sex, male	52,292 (61.1)	916 (74.4)	51,376 (60.9)	<0.001
Witnessed arrest	36,174 (42.3)	1004(81.6)	35,170 (41.7)	<0.001
Witnessed by EMS	5771 (6.7)	299(24.3)	5472 (6.5)	<0.001
Witnessed by lay rescuer	30,403 (35.5)	705 (57.3)	29,698 (35.2)	<0.001
Arrest location at public place	9203 (10.8)	300 (24.4)	8903 (10.6)	<0.001
Bystander CPR	10,555 (12.3)	347 (28.2)	10,208 (12.1)	<0.001
Initial rhythm				<0.001
PEA	5535 (6.5)	170 (13.8)	5365 (6.4)	
asystole	80,067 (93.5)	1061 (86.2)	79,006 (93.6)	
Presumed cardiac origin	79,579 (93.0)	1159 (94.2)	78,420 (92.9)	0.106
Coronary reperfusion treatment	1123 (1.3)	368 (29.9)	755 (0.9)	<0.001
TTM	1593 (1.9)	220 (17.9)	1373 (1.6)	<0.001
ECMO	410 (0.5)	20 (1.6)	390 (0.5)	<0.001
Pre-hospital ROSC	1949 (2.3)	734 (59.6)	1215 (1.4)	<0.001
Conversion	15,289 (17.9)	495 (40.2)	14,794 (17.5)	<0.001

Abbreviations; OHCA, out-of-hospital cardiac arrest; IQR, interquartile ranges; EMS, emergency medical services; CPR, cardiopulmonary resuscitation; PEA, pulseless electrical activity; TTM, targeted temperature management; ECMO, extracorporeal membrane oxygenation; ROSC, return of spontaneous circulation; CPC, cerebral performance category.

**Table 2:** Multiple logistic regression analysis for good neurologic outcome at hospital discharge.

Variable	Good Neurologic Outcome at Hospital Discharge			
	Adjusted OR	95% CI		*p*-Value
Age (years)	0.959	0.955	0.963	<0.001
Sex, male	1.230	1.055	1.434	0.008
Witnessed arrest	3.413	2.904	4.010	<0.001
Initial rhythm (PEA)	1.153	0.941	1.414	0.170
Arrest location at public place	1.422	1.204	1.680	<0.001
Bystander CPR	1.457	1.242	1.709	<0.001
Pre-hospital ROSC	72.777	63.182	83.828	<0.001
Conversion				
Conversion group	2.604	2.248	3.015	<0.001
Non-conversion group	reference			

Abbreviations; PEA, pulseless electrical activity; CPR, cardiopulmonary resuscitation; OR, odds ratio; CI, confidence interval; ROSC, return of spontaneous circulation.

**Table 3:** Resuscitation variables and outcome according to conversion to shockable rhythm in the study population.

Variable	Conversion Group (*n* = 15,289)	Non-Conversion Group (*n* = 70,313)	*p*-Value
Age (year ± SD)	65.25 ± 14.87	71.32 ± 14.86	<0.001
Age group (years)			<0.001
18~44	1381(9.0)	3901(5.5)	
45~64	5500(36.0)	16328(23.2)	
65~74	3629(23.7)	15134(21.5)	
≥75	4779(31.3)	34950(49.7)	
Sex, male	10516(68.8)	41776(59.4)	<0.001
Witnessed arrest	8583(56.1)	27591(39.2)	<0.001
Witnessed by EMS	1343(8.9)	4428(6.3)	<0.001
Witnessed by lay rescuer	7240 (47.4)	23163 (32.9)	<0.001
Arrest location at public place	2948(19.3)	2385(10.9)	<0.001
Bystander CPR	2493(16.3)	8062(11.5)	<0.001
Initial rhythm			<0.001
PEA	1353(8.8)	4182(5.9)	
Asystole	13936(91.2)	66131(94.1)	
Presumed cardiac origin	14453(94.5)	65126(92.6)	<0.001
Coronary reperfusion treatment	579(3.8)	226(1.0)	<0.001
TTM	540(3.5)	1053(1.5)	<0.001
ECMO	222(1.5)	188(0.3)	<0.001
Pre-hospital ROSC	414(2.7)	1535(2.2)	<0.001
Survival discharge	997(6.5)	2030(2.9)	<0.001
CPC 1–2	495(3.2)	736(1.0)	<0.001

Abbreviations; OHCA, out-of-hospital cardiac arrest; SD, standard deviation; IQR, interquartile ranges; EMS, emergency medical services; CPR, cardiopulmonary resuscitation; PEA, pulseless electrical activity; TTM, targeted temperature management; ECMO, extracorporeal membrane oxygenation; ROSC, return of spontaneous circulation; CPC, cerebral performance category.

**Table 4:** Resuscitation variables and outcome according to pre-hospital ROSC status in the study population.

Variable	Pre-Hospital ROSC(+)			Pre-Hospital ROSC(-)		
	CPC 1–2 (*n* = 734)	CPC 3–5 (*n* = 1215)	*p*-Value	CPC 1–2 (*n* = 497)	CPC 3–5 (*n* = 83156)	*p*-Value
Age (year), median (IQR)	56 (49–66)	67(55–78)	<0.001	56(47–66)	74(60–81)	<0.001
Age group (years)			<0.001			<0.001
18~44	101 (13.8)	108 (8.9)		97 (19.5)	4976 (6.0)	
45~64	423 (57.6)	444 (36.5)		258 (51.9)	20703 (24.9)	
65~74	130 (17.7)	240(19.8)		86 (17.3)	18307(22.0)	
≥75	80 (10.9)	423 (34.8)		56 (11.3)	39170 (47.1)	
Sex, male	548 (74.7)	776 (63.9)	<0.001	368 (74.0)	50600(6.8)	<0.001
Witnessed arrest	596 (81.2)	822 (67.7)	<0.001	408(82.1)	34348 (41.3)	<0.001
Witnessed by EMS	142(19.3)	206(17.0)	0.200	157(31.6)	5266(6.3)	<0.001
Witnessed by lay rescuer	454 (61.9)	280 (38.1)	<0.001	251 (50.5)	29082 (35.0)	<0.001
Arrest location at public place	193 (26.3)	188 (15.5)	<0.001	107 (21.5)	8715 (10.5)	<0.001
Bystander CPR	229 (31.2)	233 (19.2)	<0.001	118 (23.7)	9975 (12.0)	<0.001
Initial rhythm			0.301			<0.001
PEA	105 (14.3)	196 (16.1)		65 (13.1)	5169(6.2)	
asystole	629 (85.7)	1019 (83.9)		432 (86.9)	77987 (93.8)	
Cardiac etiology	690 (94.0)	970 (79.8)	<0.001	469 (94.4)	77450 (93.1)	0.327
Conversion	248 (33.8)	166 (13.7)	<0.001	247 (49.7)	14628 (17.6)	<0.001
Survival discharge	734 (100)	336 (27.7)	-	497 (100)	1460 (1.8)	-

Abbreviations; ROSC, return of spontaneous circulation; IQR, interquartile ranges; EMS, emergency medical services; CPR, cardiopulmonary resuscitation; PEA, pulseless electrical activity; CPC, cerebral performance category.

**Table 5:** Multiple logistic regression analysis for outcomes in OHCA patients with initial non-shockable rhythm according to pre-hospital ROSC.

Variable	Patients with Pre-Hospital ROSC (*n* = 1949)		Patients without Pre-Hospital ROSC (*n* = 83,653)	
	Survival at Discharge	CPC 1 or 2 at Discharge	Survival at Discharge	CPC 1 or 2 at Discharge
	Adjusted OR (95% CI)	Adjusted OR (95% CI)	Adjusted OR (95% CI)	Adjusted OR (95% CI)
Age (year)	0.974 (0.967–0.980)	0.968 (0.962–0.975)	0.971 (0.968–0.973)	0.954 (0.948–0.959)
Sex, male	1.105 (0.900–1.358)	1.302 (1.045–1.623)	1.105 (0.999–1.223)	1.170 (0.952–1.438)
Witnessed arrest	1.512 (1.221–1.872)	1.872 (1.479–2.369)	4.355 (3.905–4.858)	5.313 (4.203–6.716)
Initial rhythm (PEA)	0.845 (0.650–1.099)	0.882 (0.669–1.163)	2.011 (1.768–2.288)	1.469 (1.122–1.922)
Arrest location at public place	1.386 (1.079–1.781)	1.462 (1.145–1.867)	1.603 (1.425–1.804)	1.364 (1.091–1.704)
Bystander CPR	1.424 (1.126–1.800)	1.477 (1.118–1.885)	1.365 (1.219–1.529)	1.406 (1.135–1.741)
**Conversion**				
Conversion group	2.827 (2.171–3.680)	2.325 (1.821–2.935)	1.549 (1.403–1.711)	2.607 (2.170–3.131)
Non-conversion group	reference		reference	

Abbreviations; OHCA, out-of-hospital cardiac arrest; ROSC, return of spontaneous circulation; OR, odds ratio; CI, confidence interval; PEA, pulseless electrical activity; CPR, cardiopulmonary resuscitation; CPC, cerebral performance category.

**Table 6:** Multiple logistic regression analysis for outcomes in OHCA patients with initial non-shockable rhythm without pre-hospital ROSC according to the state of being witnessed by an EMS provider.

Variable	Adjusted OR (95% CI) for CPC 1 or 2 at Discharge in Patients without Pre-Hospital ROSC	
	Patients with Witnessed by EMS Provider (*n* = 5423)	Patients with Un-Witnessed by EMS Provider (*n* = 78,230)
Age (year)	0.954 (0.944–0.963)	0.954 (0.948–0.961)
Sex, male	1.052 (0.749–1.477)	1.297 (0.996–1.688)
Arrest witnessed by lay rescuer	-	3.573 (2.787–4.581)
Initial rhythm (PEA)	1.222 (0.805–1.855)	1.326 (0.929–1.892)
Arrest location at public place	0.508 (0.159–1.623)	1.812 (1.423–2.306)
Bystander CPR	1.204 (0.805–1.855)	1.309 (1.001–1.712)
Rhythm conversion		
Conversion group	1.052 (0.732–1.511)	3.972 (3.167–4.983)
Non-conversion group	reference	

Abbreviations; OHCA, out-of-hospital cardiac arrest; ROSC, return of spontaneous circulation; OR, odds ratio; CI, confidence interval; PEA, pulseless electrical activity; CPR, cardiopulmonary resuscitation; CPC, cerebral performance category.

## Data statement

The data we used were obtained from the Out-of-Hospital Cardiac Arrest Surveillance, 2012, Korea Centers for Disease Control and Prevention.
